# Neural Sensitivity to Absolute and Relative Anticipated Reward in Adolescents

**DOI:** 10.1371/journal.pone.0058708

**Published:** 2013-03-27

**Authors:** Jatin G. Vaidya, Brian Knutson, Daniel S. O'Leary, Robert I. Block, Vincent Magnotta

**Affiliations:** 1 Department of Psychiatry, University of Iowa Carver College of Medicine, Iowa City, Iowa, United States of America; 2 Department of Psychology, Stanford University, Stanford, California, United States of America; 3 Department of Radiology, University of Iowa Carver College of Medicine, Iowa City, Iowa, United States of America; 4 Department of Anesthesia, University of Iowa Carver College of Medicine, Iowa City, Iowa, United States of America; University of Missouri-Kansas City, United States of America

## Abstract

Adolescence is associated with a dramatic increase in risky and impulsive behaviors that have been attributed to developmental differences in neural processing of rewards. In the present study, we sought to identify age differences in anticipation of absolute and relative rewards. To do so, we modified a commonly used monetary incentive delay (MID) task in order to examine brain activity to relative anticipated reward value (neural sensitivity to the value of a reward as a function of other available rewards). This design also made it possible to examine developmental differences in brain activation to absolute anticipated reward magnitude (the degree to which neural activity increases with increasing reward magnitude). While undergoing fMRI, 18 adolescents and 18 adult participants were presented with cues associated with different reward magnitudes. After the cue, participants responded to a target to win money on that trial. Presentation of cues was blocked such that two reward cues associated with $.20, $1.00, or $5.00 were in play on a given block. Thus, the relative value of the $1.00 reward varied depending on whether it was paired with a smaller or larger reward. Reflecting age differences in neural responses to relative anticipated reward (i.e., reference dependent processing), adults, but not adolescents, demonstrated greater activity to a $1 reward when it was the larger of the two available rewards. Adults also demonstrated a more linear increase in ventral striatal activity as a function of increasing absolute reward magnitude compared to adolescents. Additionally, reduced ventral striatal sensitivity to absolute anticipated reward (i.e., the difference in activity to medium versus small rewards) correlated with higher levels of trait Impulsivity. Thus, ventral striatal activity in anticipation of absolute and relative rewards develops with age. Absolute reward processing is also linked to individual differences in Impulsivity.

## Introduction

Adolescence is marked by a dramatic increase in risky, impulsive behaviors that are associated with significant morbidity and mortality [1] Individual differences on trait measures of Impulsivity peak during adolescence and decline thereafter into young adulthood [2], [3]. Immaturities in frontal lobe executive control systems have been posited to contribute to problems with impulse control during adolescence [4], [5]. However, shifts in the activity of dopamine modulated incentive motivational circuitry may additionally alter approach-oriented behaviors [6]. Animal research, for instance, has shown that dopaminergic receptor density in the striatum is highest during adolescence [7]. Additionally, peri-adolescent rats show greater sensitivity to dopamine antagonists (e.g., haloperidol) but less sensitivity to dopamine agonists than younger and older animals [8].

Many human developmental neuroimaging studies have focused on determining whether adolescents demonstrate hyper- or hypo-sensitivity to monetary rewards compared to adults [9]–[13]. These studies have sometimes reported disparate findings. For instance, using variations of the widely-used monetary incentive delay (MID) task, Bjork and colleagues reported reduced activity in the ventral striatum (VS) in adolescents compared to adults during reward anticipation [9], [14]. On the other hand, Galvan and colleagues [11], using a somewhat different monetary reward task, found evidence for adolescent hyper-activity in the VS in response to reward outcomes. Geier and colleagues [12] using an elegantly designed protocol that parsed distinct phases of reward processing (reward cue assessment, response preparation/anticipation, and behavioral response), demonstrated that the exact pattern of developmental differences depended on the specific phase of reward processing under examination. In their study, adolescents demonstrated lower VS activity during cue assessment but higher VS activity during behavioral responses [12].

Although the timing of neural signals may explain some of the discrepant results across studies, a number of other important questions remain regarding developmental differences in reward sensitivity. For instance, in adult subjects, Knutson and colleagues have demonstrated that the blood oxygen level dependent (BOLD) response in the VS increases proportional to the magnitude of anticipated reward during the MID task [15]. Adults demonstrate a near-linear increase in BOLD activity during reward anticipation as reward value increases from $.20 to $1.00 to $5.00. Using the MID task in adolescents, however, Bjork and colleagues (their Figure 2b) did not observe a significant increase in VS activity in conjunction with increasing reward magnitude [9], although they did not formally test this effect.

**Figure 2:**
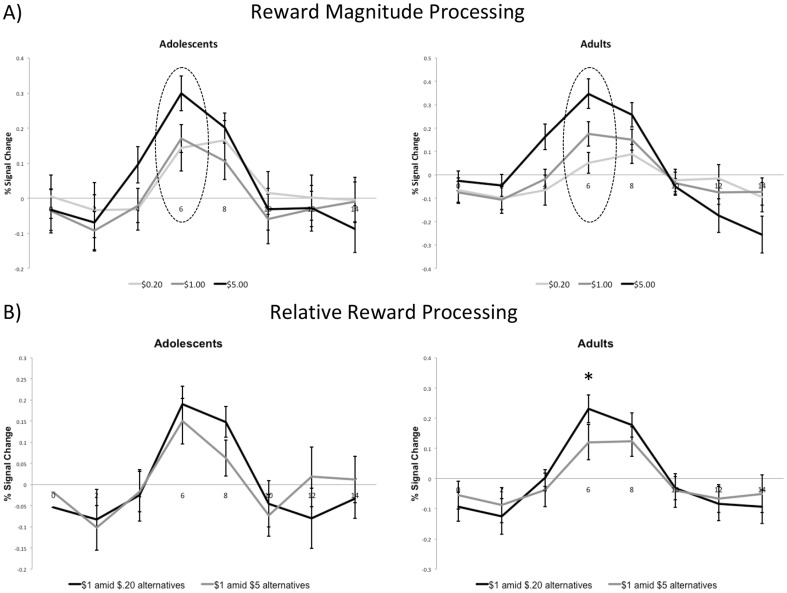
Group differences in reward processing in the right VS. A) Effect of absolute reward magnitude processing. Adults compared to adolescents demonstrated a more linear increase in anticipatory reward activation as a function of absolute reward magnitude at the point of peak activation (6 seconds post-stimulus onset; p = .02). B) Relative reward activity in VS to $1 reward. Based on paired t-tests for each age group, results showed that adults (p = .007) but not adolescents (p = .34) demonstrated significant differences in VS activity at 6 seconds post-stimulus onset to the $1 reward depending on the relative context.

Previous studies of adolescents have also not examined relative reward processing, or differential processing of rewards in the context of other available rewards. In adult animals, dopamine neuron firing tracks with the subjective value rather than physical properties of a stimulus [16]. For instance, in one study, the same dopamine neuron that demonstrated limited activity to a moderately preferable reward (apple) when paired with a more preferable reward (raisin) showed much higher activity when the moderate reward was paired with a less preferable reward (cereal) [17]. This sort of reference dependency is a central tenet of prospect theory [18] and facilitates effective decision making since humans have difficulty making absolute, reference independent judgments [19] fMRI studies in adult humans broadly suggest that the relative value of a stimulus modulates activity in dopamine rich regions such as the orbitofrontal cortex [20] and striatum [21] although the VS appears to be particularly strongly linked to relative/reference-dependent valuations [19]. However, research has not examined whether adolescents demonstrate similar patterns of relative reward activations. Whereas overall differences between adolescents and adults may reflect general insensitivity to absolute rewards, insensitivity to relative reward value may reflect a decrement in the ability to adequately contextualize the value of a reward given other available alternatives. Given that the availability of rewards changes over time, the ability to recalibrate the value of a given reward in reference to other available rewards may depend on appropriate development of reward circuitry and may ultimately improve decision-making. In fact, one study found that individuals with autism spectrum disorder, a developmental disorder, fail to use emotional context during decision-making [22].

Furthermore, if adolescents fail to show relative reward activation effects, then they may appear to be hyper- or hypo-sensitive to rewards compared to adults depending on other available rewards. Thus, apparent developmental differences in hyper- or hypo-activations might possibly reflect differences in relative reward valuation. Finally, while a number of studies have examined relations between brain activity to rewarding stimuli and individual differences in Impulsivity [23]–[26], there has been limited systematic investigation of the association between different aspects of reward processing and Impulsivity in a developmental context. Examining absolute and relative reward-Impulsivity associations marks a critical step towards determining whether developmental shifts in reward processing influence adolescent Impulsivity.

In this study, we examined whether adolescents differ in neural responses to relative and absolute anticipated reward. In order to identify differences in relative reward activation, we modified the canonical MID task in a manner that made it similar to a relative reward study in monkeys [17]. Thus, in the present study, we were able to determine if activity to a $1 reward cue varied depending on whether it was the preferred of two available alternative rewards (i.e., $.20 vs. $5.00) during a given block of trials. Given earlier findings of blunted VS activity in adolescents [9], we predicted that adolescents, compared to adults a) would demonstrate less of an increase in anticipatory reward BOLD activity as a function of absolute reward magnitude and b) would also demonstrate reduced activity during anticipation of relative reward in the VS. Also, because earlier studies have shown that the mesial PFC/orbitofrontal PFC neurons are sensitive to relative reward value [17], we conducted exploratory analyses to examine developmental differences in mesial PFC activations. Given that earlier studies have tended to emphasize anticipatory reward differences in the VS as oppose to outcome activity in the mesial PFC [9], [14], we did not make specific hypotheses about developmental differences in this region. Finally, we also conducted exploratory analyses that examined associations between individual differences in personality traits related to Impulsivity as a function of anticipatory activity in the VS. Individual differences in traits related to Impulsivity have been associated with reduced VS activity to anticipatory reward activity in healthy individuals [23], [24], [27]. However, no studies to date have examined associations between Impulsivity and absolute or relative reward anticipatory activity. In the context of adolescence, characterizing associations between VS activity and Impulsivity may help to account for shifts in Impulsivity over development.

## Methods

### Participants

Participants consisted of adolescents 12 to 15 years of age and young adults 26 to 30 years of age. These age ranges are largely consistent with previous studies on adolescent reward processing (e.g., [9], [11]). All participants were right handed and had no history of neurological problems (e.g., extended loss of consciousness, seizures, stroke) or learning/developmental disorders. Participants were free from psychiatric disorders as determined by a structured clinical interview conducted by a trained research assistant (Comprehensive Assessment of Symptoms and History) [28]. Informed consent was obtained for adult subjects and parental consent and subject assent were obtained for adolescents. The study procedures were approved by the University of Iowa Institutional Review Board. One adolescent subject from the initial sample was excluded due to excessive head motion in the MRI scanner. The final sample consisted of 18 adolescents (9 females; mean age = 13.39, SD = .92) and 18 young adults (9 females; mean age = 27.72; SD = 1.36). Participants were compensated $100 for taking part in the study and also were paid the average amount they won on the three fMRI runs (approximately $25).

### Modified MID task

Participants took part in three runs of a modified MID task. The original MID task developed by Knutson and colleagues consists of gain (reward), loss, and neutral cues that precede a button press response to a white square target [29]. If the subject successfully responds with a button press during target presentation, he or she gains (reward trials) or avoids losing money (loss trials). The period between and after target presentation is variable but constrained so that the reward outcome notification is provided 4250 ms after cue offset. Whereas individual cues were presented in a purely event-related manner in the original version of the MID task, cue presentation was modified to a mixed blocked and event-related design for the present study. Each run consisted of three blocks. In a given block, two of three rewards were cued. For instance, in block A, the $.20 reward (corresponding with a circle cue with one line) and the $1.00 reward (corresponding with a circle cue with two lines) were presented. Block B consisted of $1.00 and $5.00 rewards and Block C consisted of $.20 and $5.00 rewards. Block order was counterbalanced across runs and subjects (however, block C was always presented last in the run because this block was of limited interest; see below for more information). Each block within a given run consisted of 18 trials (9 small and 9 large rewards). Trials were separated by a constant inter-trial interval of 4 seconds. Prior to the beginning of each block, subjects saw a screen that informed them as to which of the two cues would be presented during that block. Total scanning time for each of the three runs was 600 seconds. As in the original MID task, task difficulty was set based on practice task performance such that each participants could succeed in “hitting” targets on approximately 66% of all trials. Unlike the original MID task, only reward cues were included in this modified version in order to limit scanning time and to adequately power comparisons between different reward cues.

### Impulsivity measures

As part of a larger battery of questionnaires, participants completed two widely used self-report measures of personality broadly related to the trait of Impulsivity. The Barratt Impulsiveness Scale (BIS-11; [30]) consists of three sub-scales: Attentional Impulsiveness, Motor Impulsiveness, and Non-planning Impulsiveness. The NEO-PI-R [31] Conscientiousness domain consists of 6 facets: Competence, Order, Dutifulness, Achievement Striving, Self-discipline, and Deliberation (these scales were reverse coded so that higher scores reflect greater Impulsivity). Based on previous factor analytic work, scores on BIS-11 and NEO-PI-R Conscientiousness facet scales were combined in the following manner to create two Impulsivity composite indices: low Accomplishment (A_Imp; consisting of Achievement Striving, Self-discipline, Competence, Dutifulness, Non-planning Impulsiveness, and Order) and low Self-Control (S_Imp; consisting of Deliberation and Motor Impulsiveness) [3]. Low Accomplishment reflects a lack of perseverance and an absence of significant long-term goals, whereas Low Self-Control reflects a lack of premeditation and a failure to appreciate the long-term consequences of one's behavior [3], [32].

### Magnetic resonance imaging parameters

Imaging was performed on a 3T Siemens (Erlangen, Germany) Trio MRI scanner equipped with a 12-channel head coil. Twenty seven 3.75 mm thick slices (including a .75 mm gap; in plane resolution = 3.75 mm×3.75 mm) were acquired using a T2* sensitive echoplanar imaging sequence (TR = 2000 ms, TE = 30 ms, flip angle = 90°). Images were collected at 30° from the AC-PC line to reduce signal dropout artifact in the ventral forebrain (Deichmann, 2003. An MP-RAGE T1 (TR = 2300 ms, TE = 2.82 ms, flip angle = 10 degrees, FOV = 282×282×264) structural scan was also acquired for functional coregistration.

### Image preprocessing

Functional imaging analyses were conducted using Analysis of Functional Neuroimages (AFNI) software [33]. Individual subject data were corrected for slice time offset in order to correct for temporal differences in slice acquisitions. Outliers within a voxel timeseries were replaced using a despiking algorithm. fMRI data were motion corrected by fitting all images to a single base image which corresponded to the volume that was most similar to the median image (using 3dTqual) for that subject; inspection of motion correction data revealed that no subject had moved by more than 2 mm from one volume to another in any direction. A relatively small spatial smoothing kernel (4 mm at FWHM) was applied on the EPI data because recent findings have shown that larger degrees of smoothness can systematically bias localization of activations in the area of the VS [34]. Temporal smoothing was conducted to remove low frequency fluctuations (<.011 Hz) following Bjork and colleagues [9].

### Statistical analyses

The overall analytic strategy was to investigate group differences in activity as a function of relative and absolute reward magnitude. To do so, we utilized both a region of interest (ROI) approach that focused on the VS and mesial PFC (see below for details) and an exploratory whole brain analysis approach.

#### ROI analyses

For the ROI analyses, we estimated the time course of activity at the single subject level using a regression model that included the following regressors of interest for both hit and miss outcomes (i.e., 12 regressors of interest): a) $.20 in Block A, b) $1.00 in Block A, c) $1.00 in Block B, d) $5.00 in Block B, e) $.20 in Block C, f) $5.00 in Block C. For the VS, hit and miss time courses for a given reward level/Block were aggregated. Because we were primarily interested in reward activations on hit outcomes in the mPFC and because there were many more hit compared to miss trials (by design), mPFC time courses only included hit trials. This way, we were able to determine if activity in mPFC during reward outcome processing varied as a function of absolute or relative value. We did, however, plot the time courses for the miss trials and found that the basic pattern of results for these trials was highly similar to the hit trials. The shape of the hemodynamic response was modeled using a set of 8 cubic spline basis functions resulting in a 14 second time course for each reward trial. Impulse response functions generated from these analyses were warped to the template image as described below in section 2.6.2. Left and right VS and mesial PFC ROIs were defined based on reward activation results previously reported by Bjork and colleagues (2010) in a study of healthy adolescents and adults using a variation of the original MID task. 5 mm radius spheres were centered at +/−6, 8, 0 for the left/right VS and 6, 54, −6 for the mesial PFC. After identifying the time point that showed peak anticipatory reward activity (typically about 4 to 6 seconds into the impulse response) and reward outcome activity (typically about 8 to 10 seconds into the impulse response) [29], we examined that peak activity as a function of relative value and reward magnitude. Additionally, we conducted correlational and regression analyses in order to identify associations between absolute magnitude activity and relative reward activity as a function of age and Impulsivity.

#### Whole brain analyses

Preprocessed time series data were analyzed using general linear modeling at the individual subject level. Regressors for the whole brain analyses were set up as contrasts, such that the general linear model explicitly included absolute and relative gain anticipation regressors as well as an absolute outcome regressor. In the absolute gain anticipation regressor, the 2-second period following the $.20 cue was coded as a −1, the $1.00 reward cue was coded as a 0, and the $5.00 reward cue was coded as +1 regardless of block. In the relative gain anticipation regressors, the smaller of the two rewards within a block was coded as a −1 and the larger of the two rewards was coded as +1. In the absolute outcome regressor, “hits” were coded as +.20, +1.0, +5.0 and “misses” were coded as −.20, −1.0, and −5.0. Thus, absolute and relative anticipation regressors were orthogonalized by differential weighting of the $1.00 reward. All regressors were convolved against a gamma variate impulse response function, which provides a good estimate of the hemodynamic response across age groups [35]. In addition to the regressors of interest, six motion regressors of no interest were also included in the model. The beta values for regressors were coregistered to each individual's T1 image using a rigid coregistration algorithm. The T1 image was warped to a 152 subject average Talairach template structural image using a 12-parameter warping algorithm and the resulting transform was applied to the functional images to warp these images to template space (voxels were resampled to 2 mm isotropic). Whole brain group analyses were conducted in AFNI on the coefficients associated with each regressor warped to template space. Two-group t-tests were conducted on each of the three regressors. Correction for multiple comparisons was implemented using a combined voxel-wise and cluster-size threshold. Based on the estimated spatial smoothness of the data (which was derived using AFNI's 3dFWHMx; smoothness in the x, y, and z, planes were 6.6, 6.2, and 5.5, respectively), Monte Carlo simulations were run using AFNI's 3dAlphaSim. With an uncorrected p-value set to .005, these simulations resulted in a cluster size of 89 voxels in order to achieve a corrected alpha of .05.

## Results

### Behavioral findings

**Figure 1:**
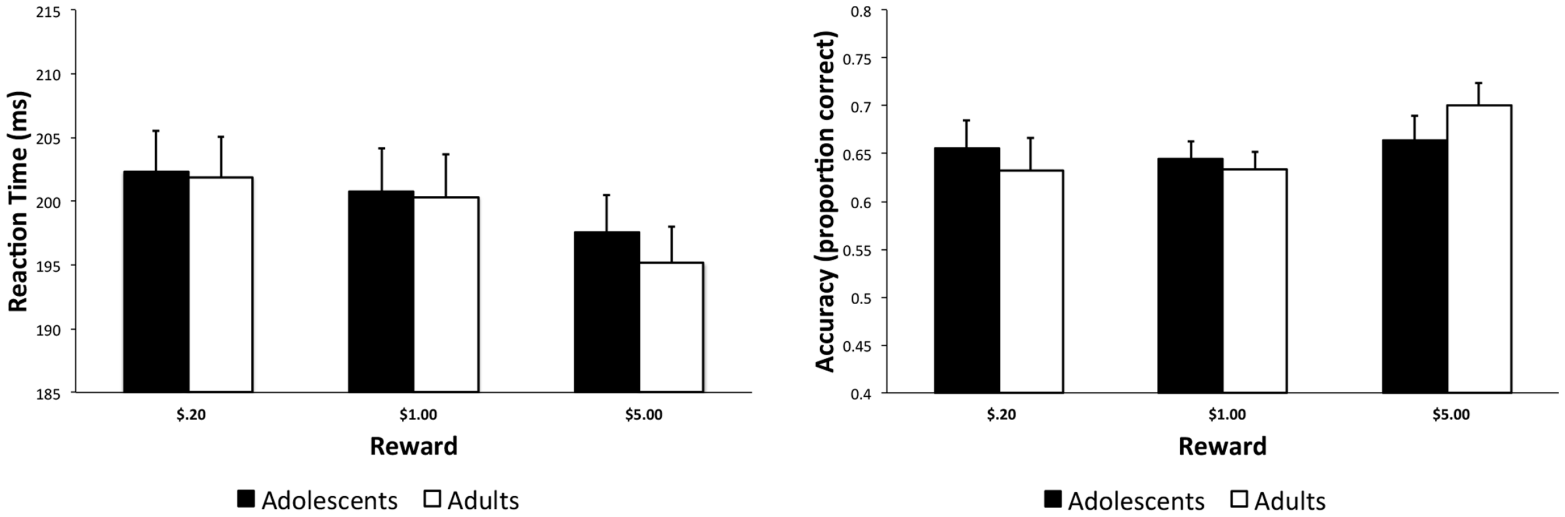
Behavioral performance on the modified MID Task. As described in greater detail in the text, adults and adolescents were faster and more accurate on high reward trials.

The modified MID task, like the original MID task, is designed to control for performance by adaptively adjusting the target presentation period from run to run. To verify the effectiveness of this adaptive procedure and to ensure that the groups did not differ on behavioral performance, ANOVA was conducted on hit rate (proportion of hit responses) and reaction time (hit trials only). A 2 (Age group: young, old) X 3 (Reward: $.20, $1.00, $5.00) ANOVA collapsed across blocks revealed a marginally significant main effect of reward on accuracy [F(2, 68) = 2.69, p = .08]. Importantly, the main effect for age group was not significant (p = .97) nor was the age group X reward interaction effect (p = .30). For reaction time, the main effect for reward was significant [F(2, 68) = 19.12, p<.001] but the effects for age group (p = .80) and the age group X reward interaction (p = .49) were not. As shown in Figure 1, both groups showed a trend towards greater accuracy and faster reaction time on high reward trials. When examining performance on the $1.00 reward trials in Block A vs. Block B in a 2 (Age group) X 2 (Block) ANOVA, there were no main effects for block or age group, nor was there a block X Age group interaction effect for hit rate or reaction time (ps>.21).

### ROI analyses

**Figure 3:**
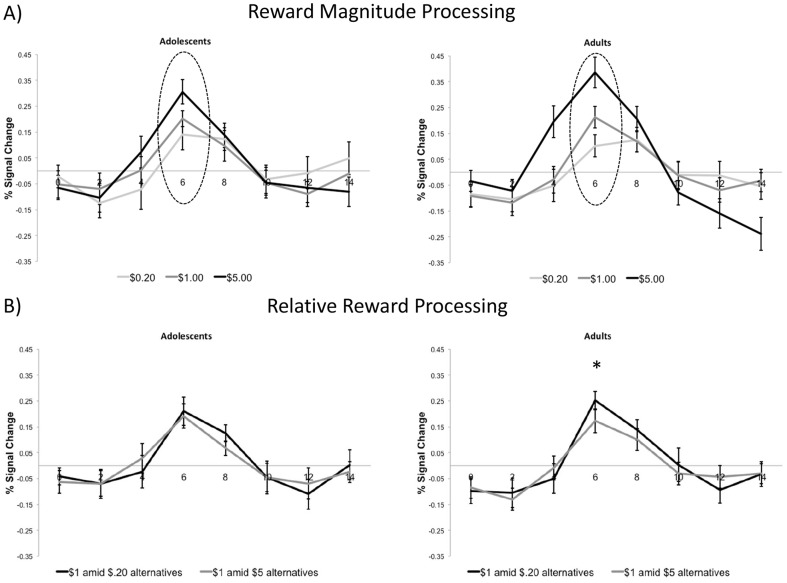
Group differences in reward processing in the left VS. A) Effect of absolute reward magnitude processing. There was a trend for adults compared to adolescents to demonstrate a more linear increase in anticipatory reward activation as a function of absolute reward magnitude at the point of peak activation (6 seconds post-stimulus onset; p = .07). B) Relative reward activity in VS to $1 reward. Based on paired t-tests for each age group, results showed that adults showed greater activity at 6 seconds post-stimulus onset in the $1 amid $.20 alternatives condition (p = .01). The same effect was not significant for adolescents (p = .65).

Time courses for the VS ROIs are presented in Figure 2 and 3. The time courses clearly show a peak in activation at 6 seconds for the left and right ROI, corresponding to anticipatory reward activity. The following analyses in the VS thus focus on activation at this point. Also, as noted earlier, time courses shown in Figures 2 and 3 include both hit and miss trials (see Methods above for details). For the right VS, collapsing across reward context in order to investigate the impact of absolute reward magnitude as a function of age group, ANOVA revealed that there was no main effect of age group [F(1, 34) = .04, p = .84], thus indicating that both groups showed similar levels of activity in the VS. As expected, the main effect of reward magnitude was highly significant [F(2, 68) = 43.72, p<.001]. There was also a significant interaction between magnitude and age group [F(2, 68) = 4.27, p = .02]. As shown in Figure 2A, adults' peak VS activation discriminated more between different absolute reward magnitudes. Next, we examined activity to the $1 reward as a function of relative reward context in the right VS (see Figure 2B). As predicted, adults [t(17) = 3.09, p = .007] but not adolescents [t(17) = .98, p = .34] demonstrated differences in anticipatory activity to the medium, $1 reward depending on the relative context of the reward. The interaction between relative reward value and age group did not reach conventional levels of significance for the right VS [F(1, 34) = 1.70, p = .20].

Results for the left VS were largely consistent with the right VS results (Figure 3). For absolute reward anticipation in the left VS, there was no main effect of age group [F(1, 34) = .01, p = .76] but there was a main effect for reward magnitude [F(2, 68) = 38.99 = p<.001]. There was a nearly significant interaction effect between age group and reward magnitude [F(2, 68) = 2.71, p = .07] (see Figure 3A). Consistent with the relative reward anticipation finding in the right VS, adults [t(17) = 2.81, p = .01] but not adolescents [t(17) = .47, p = .65) showed significant differences in activity based on relative reward value (see Figure 3B). The interaction between relative reward value and age group did not reach conventional levels of significance for the left VS [F(1, 34) = 1.44, p = .24].

**Figure 4:**
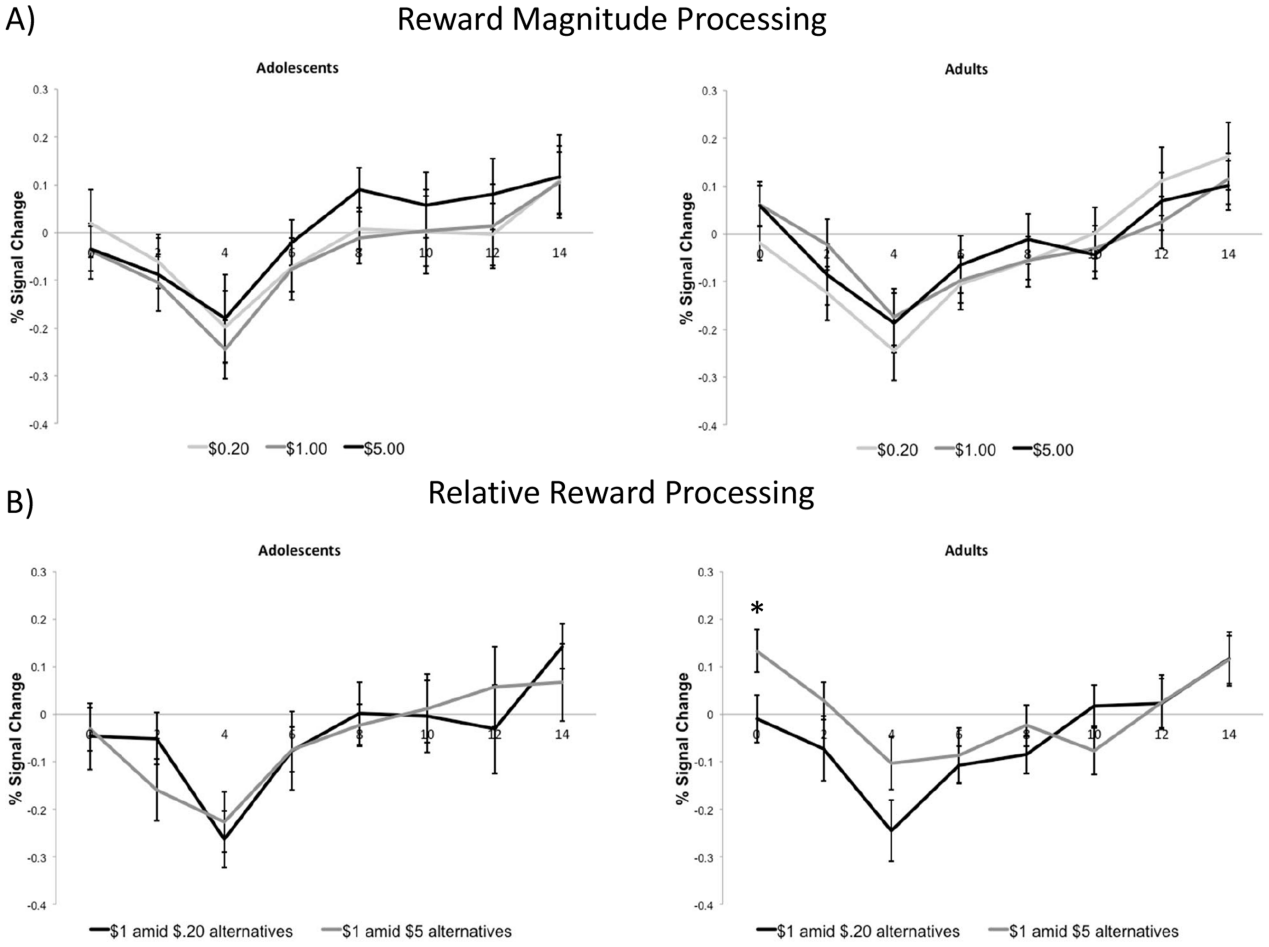
Group differences in reward processing in the mesial PFC. Only hit trials were included for mesial PFC time courses. A) Effect of absolute reward magnitude processing in mesial PFC. There was a main effect at 8 s as a function of absolute reward value (p = .01). However, there were no significant interaction effects between absolute reward value and age group at any time point (ps>.05). B) Relative reward activity to the $1 reward in mesial PFC. A paired t-test indicated that adults did show a difference in the degree to which mesial PFC activity decreased as a function of relative reward value at Time 0 (p = .01). However, none of the interaction effects between relative reward value and age group were significant at any time point (ps>.05).

In the mesial PFC ROI, Figure 4A presents the time courses for adolescent and adult age groups as a function of absolute reward magnitude (for “hit” only trials). For these analyses, we were primarily focused on the later, reward outcome related activation findings (approximately corresponding to 8 to 10 s post-cue). There was a main effect at 8 s as a function of absolute reward value [F(2, 68) = 4.99, p = .01] but there were no other main effects at any of the other time points. Additionally, there were no significant interaction effects between absolute reward value and age group (ps>.05). Figure 4B presents reward activation patterns for the two groups as a function of relative value for the $1.00 reward. There was no evidence that mesial PFC activation varied during the outcome phase as a function of relative reward value. Additionally, none of the interaction effects between relative reward value and age group were significant (ps>.05; see Figure 4B).

### Associations with impulsivity

Consistent with existing findings [3], adolescents scored higher on A_Imp [t(34) = −2.50, p = .02] and demonstrated a trend towards scoring higher on S_Imp [t(34) = −1.71, p = .10]. We examined the association between two indices of Impulsivity used in this study with anticipatory activation in the left and right VS at the 6 second time point, which represents the peak of the anticipatory activation period. In order to derive an individual differences measure of reward magnitude sensitivity, we created two new variables—one representing the difference in activity between anticipation of the $1.00 and $.20 reward, and another representing the difference in activity between anticipation of the $5.00 and $1.00 rewards. All subjects were included in these analyses but age group was entered as a control variable. Thus, the correlations in Table 1 reflect associations between Impulsivity and net difference in VS activity after controlling for age group. Spearman rank-order correlations revealed that both measures of Impulsivity were negatively correlated with the $1.00 vs. $.20 contrast indicating that individuals who scored higher on Impulsivity showed less differentiation in VS activity between the $1.00 and $.20 rewards. Although not statistically significant, there was a relatively consistent trend for the correlations between Impulsivity and activity during $5.00 versus $1.00 reward anticipation to be positive (and in the opposite direction as the aforementioned correlations with the $1.00 vs. $.20 contrast). Follow-up analyses using William's modification of Hotelling's test were conducted to determine if these correlations differed significantly from one another [36], and this was the case for Right VS activity. Thus, for A_Imp, −.38 is significantly different from .22 (Z = 2.04, p<.05). For S_Imp, −.34 is significantly different from .31 (Z = 2.16, p<.05). The correlations for the left VS did not significantly differ (ps>.05).

**Table 1:** Correlations between net difference in reward activity in the VS and Impulsivity.

	Impulsivity component	
Reward contrast	A_Imp	S_Imp
*Right Ventral Striatum*		
$1 vs. $.20	−.38*	−.34*
$5 vs. $1	.22	.31
*Left Ventral Striatum*		
$1 vs. $.20	−.30	−.35*
$5 vs. $1	.13	.15

Note. Correlations reflect Spearman's rank-order correlations and control for age group.

* p<.05.

Within age group analyses were also conducted. Although the effects did not reach conventional levels of significance due to smaller sample sizes, effects remained in the same direction as in the whole group analyses. For instance, higher scores on A_Imp were associated with smaller differences between the $1.00 and $.20 reward in the right VS [r(16) = −.43, p = .08] and left VS [r(16) = −.38, p = .12] in adolescents. Adults showed similar correlations in the right VS [r(16) = −.34, p = .17] and left VS [r(16) = −.19, p = .44). Correlations between Impulsivity and relative reward anticipation activity (comparing activity to the $1 reward in the two reward contexts) were not significant (ps>.05).

### Whole brain analyses

**Figure 5:**
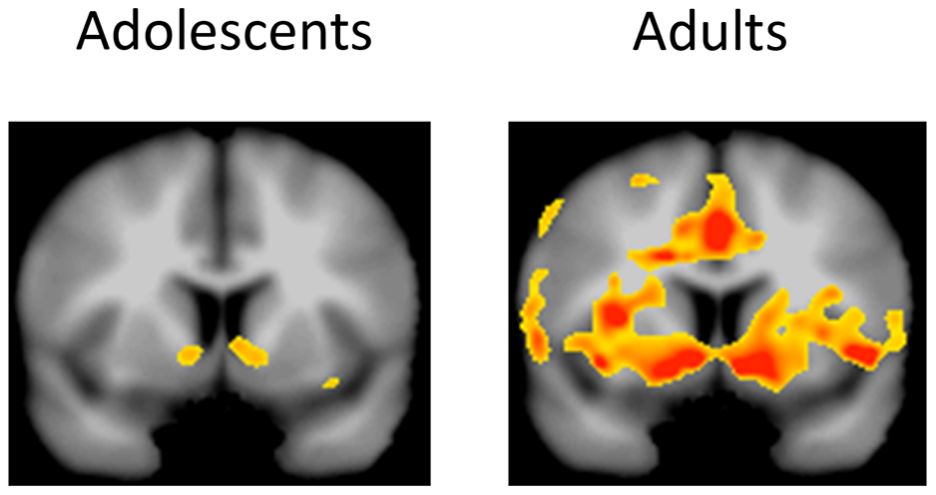
Anticipated absolute reward magnitude activity in adolescents and adults for the whole brain analyses. Results are shown on a coronal slices for the VS (y = 8). Although the direct comparison between the two groups was not significant, the within group analyses show that the adults demonstrated strong, bilateral activation in the VS and other adjacent regions whereas the effect was much weaker in the adolescents. P-value threshold set to .005 as in other whole brain analyses.

For whole brain analyses, we conducted three sets of analyses to examine within and between group differences on absolute reward anticipation, relative reward anticipation, and absolute reward outcome. For absolute anticipation, adults showed increased activity as a function of anticipated reward magnitude in a number of regions including the VS, anterior cingulate, thalamus, and insula (see Table 2). In the adolescent sample, no regions showed significantly increased activity for absolute anticipation at the whole brain correction threshold (see also Figure 5). In the direct comparison between adolescents and adults on the absolute anticipation regressor, only one region, a portion of the cuneus (BA 19; x = −24, −88, 30; peak t = 4.42; cluster size = 179), showed significant group differences such that adults showed greater absolute anticipatory reward activity than adolescents. For the relative reward regressor, adults demonstrated greater activity to the $1 reward when it was the larger of the two available rewards in a large region including the right striatum and precuneus (see Table 3). There were no significant effects for the adolescents on this regressor, and there were no significant group differences. The final set of analyses involved the absolute outcome regressor. Both age groups demonstrated robust activations to the absolute outcome regressor. As shown in Table 4, adolescents and adults demonstrated greater activity in a number of regions including the mesial PFC, anterior cingulate, cerebellum, and temporal lobe when processing rewarding outcomes as opposed to failure to win outcomes. The two-group t-test did not reveal any regions that showed significant differences in activity between adolescents and adults in response to reward outcomes.

**Table 2:** Regions demonstrating significant activity for absolute anticipation.

Region	L-R	x	y	z	Peak t	Cluster size
**Adolescents**						
–						
**Adults**						
Middle Frontal Gyrus (BA 46)	R	42	40	16	6.34	380
Rostral Anterior Cingulate (BA 32)	R	8	38	22	7.89	164
Middle Frontal Gyrus (BA 9)	L	−32	34	34	6.78	246
Dorsal Anterior Cingulate (BA 24)	M	−8	12	32	9.23	2120
Precentral Gyrus (BA 6)	R	40	−12	44	7.13	609
Superior Temporal Gyrus (BA 41)	L	−56	−28	12	6.82	396
Inferior Parietal Lobule (BA 40)	R	50	−32	24	5.01	136
Mesial Occipital lobe/thalamus/VS	M	12	−64	14	9.39	14810
Middle Occipital Gyrus (BA 19)	R	48	−70	8	5.52	141
**Adults>Adolescents**						
Cuneus (BA 19)	L	−24	−88	30	4.42	179

Note. Negative peak t-values indicate areas that showed greater activation to failure to obtain rewards. VS = ventral striatum; M = mesial.

**Table 3:** Regions demonstrating significant activity for relative anticipation.

Region	L-R	x	y	z	Peak t	Cluster size
**Adolescents**						
–						
**Adults**						
VS	R	6	10	2	5.64	232
Precuneus (BA 13)	R	28	−38	28	4.74	104
**Adults>Adolescents**						
–						

VS = ventral striatum.

**Table 4:** Regions demonstrating significant activity to amount of gain vs. failure to gain.

Region	L-R	x	y	z	Peak t	Cluster size
**Adolescents**						
Middle Frontal Gyrus (BA 46)	R	52	42	20	7.69	315
Superior Frontal Gyrus (BA 8)	R	20	30	44	8.74	243
Superior Frontal Gyrus (BA 6)	R	18	30	56	6.22	218
Ventromedial PFC/VS/Occ. Cortex	M	6	26	−16	11.24	14479
Middle Frontal Gyrus (BA 6)	L	−28	14	44	6.31	588
Middle Temporal Gyrus (BA 37)	R	58	−46	−6	6.39	232
Cerebellum (Pyramis)	R	42	−66	−36	6.34	255
Cerebellum (Pyramis)	L	−36	−68	−34	8.1	599
Middle Frontal Gyrus (BA 46)	L	−46	28	22	4.86	90
Inf. Frontal Gyrus/Insula (BA 47)	L	−34	18	−8	−4.66	101
Dorsal Anterior Cingulate (BA 24)	L	−8	12	30	−4.3	135
Insula (BA 13)	L	−32	10	8	−5.09	148
Inferior Parietal Lobule (BA 40)	R	60	−34	28	−8.06	192
**Adults**						
Rostral Anterior Cingulate (BA 32)	M	0	42	12	5.02	190
Middle Frontal Gyrus (BA 46)	L	−48	26	26	7.03	314
Superior Frontal Gyrus (BA 8)	L	−18	24	46	8.41	9257
Superior Frontal Gyrus (BA 8)	R	30	20	56	9.64	1788
Thalamus	M	0	0	6	5.86	100
Parahippocampal Gyrus	L	−20	−32	0	6.68	181
Middle Temporal Gyrus (BA 21)	L	−58	−38	−12	6.81	337
Middle Temporal Gyrus (BA 37)	R	60	−46	−8	8.84	133
Cerebellum (Tuber)	R	34	−64	−30	5.78	361
Cerebellum (pyramis)	L	−40	−72	−34	8.43	3320
Superior Occipital Gyrus (BA 19)	R	34	−86	22	5.84	426
Superior/Medial Frontal Gyrus (BA 6)	M	8	12	56	−5.48	448
Insula (BA 13)	L	−44	8	4	−6.01	202
Postcentral Gyrus (BA 3)	L	−44	−18	46	−4.80	240
Inferior Parietal Lobule (BA 40)	R	60	−42	26	−4.66	139
**Adults>Adolescents**						
–						

Note. Negative peak t-values indicate areas that showed greater activation to failure to obtain rewards. Occ. = occipital; PFC = prefrontal cortex; VS = ventral striatum; M = Mesial.

## Discussion

Combining fMRI with a modified MID task, we identified age group differences in neural activity during reward anticipation. The task modifications made it possible to identify age differences in neural activity during anticipation of absolute and relative reward magnitude. As expected, adolescents demonstrated less of a linear increase in VS activity than adults during anticipation of increasing reward magnitude. Furthermore, individual differences in anticipatory reward related VS activity were negatively correlated with individual differences in trait Impulsivity. Additionally, and in line with our novel predictions, we found preliminary evidence indicating that VS activity in adolescents was less sensitive to relative reward value. Specifically, adults, but not adolescents, demonstrated greater activity to a $1 reward when it was the preferred of the two available rewards. Both groups, however, showed a similar response to reward outcomes in the mesial PFC. Taken together, these findings point toward reduced adolescent sensitivity to anticipated reward, both in an absolute and relative sense.

### Anticipation of absolute and relative reward in adults and adolescents

In this study, adults showed evidence of neural sensitivity to anticipated relative rewards in the VS. Although earlier investigations demonstrated differences in relative reward processing [20], [21], [37], our findings extend this work by demonstrating that adults clearly discriminate relative anticipated rewards in the VS. Since VS activity has been linked to incentive motivation [38], the current findings suggest it responds to relative as well as absolute anticipated rewards in adults. Additionally, these preliminary findings suggest that there may developmental differences neural processing of relative reward value. Unlike adults, adolescents failed to show significant differences in relative reward activity. Although adults showed significant VS responses to relative anticipated rewards while adolescents did not, direct comparison of adolescents to adults did not reveal statistically significant differences, possibly due to reduced power of group comparisons. Additionally, there were no differences in behavioral responding (i.e., reaction time, accuracy) as a function of relative reward value, although this may have been because of the adaptive nature of the MID task which adjusts task difficulty from run to run. Behavioral responses that don't meet a critical threshold (adaptively scaled for a given subject) will lead to no reward outcomes. Consequently, there may not have been sufficient variability in reaction time due to the adaptive nature of the task. Thus, we report the within group findings as preliminary evidence for age differences in neural responses to relative reward valuation effects.

Further research exploring age differences in absolute and relative reward processing with different tasks is needed. The reduced anticipatory reward sensitivity observed in the present study may specifically relate to the use of a modified MID task. The few existing relevant studies suggest that adolescents are more likely to show reduced neural responses to anticipated rewards in the context of the MID task [9], [14]. If future studies that use different tasks and incentives show similar results, reduced reward anticipation in adolescents may have a number of practical implications. Importantly, if adolescents fail to explicitly incorporate relative context into their anticipation of future rewards, they may appear to be either hypo- or hyper-sensitive to rewards depending on other available rewarding alternatives. For instance, on a given task with several available rewards, adolescents may show increased activity when anticipating a small reward relative to adults because they fail to adequately contextualize its value compared to other larger rewards. Alternatively, adolescents may demonstrate reduced activity when anticipating a large reward as a function of not contextualizing its value compared to other smaller rewards. Existing findings are partially consistent with this possibility. In a MID task with three different reward magnitudes, Bjork and colleagues reported reduced VS activation during anticipation of the largest reward ($5.00) in adolescents compared to adults. However, the same study did not find group differences in VS activity during anticipation of the smallest ($0.20) reward. On the other hand, group differences may be most evident during anticipation of large (e.g., $5.00) rewards because they generate the most reliable signal in the VS. Future studies that include multiple reward levels that can all reliably elicit VS activation could best test the impact of reference dependency on neural responses during anticipation various levels of reward in adolescents and adults.

Another implication of these findings is that neural circuits that promote reward anticipation change and develop with age. For instance, developmental changes in dopamine signaling may contribute to changes in reward valuation. Studies of rats suggest that the dopamine system may be functioning at a higher baseline level in adolescence [6]. Specifically, adolescent rats compared to both younger and older animals show greater sensitivity to the dopamine antagonist haloperidol but lower sensitivity to dopamine agonists [8]. Thus, higher tonic dopamine activity may result in a less dramatic increase in phasic dopamine release and correlated fMRI activity in adolescents than adults [39], [40]. Although the fMRI blood oxygen level dependent signal does not directly index the firing of dopamine neurons, considerable animal and human evidence suggests that dopamine release in the VS can increase local fMRI activity [41].

Another neural circuit that might contribute to age differences in neural responses during reward anticipation involves projections from the mesial PFC. Since the PFC continues to mature well into the third decade of life [42]–[44], and mesial and orbital regions of the PFC are involved in reward valuation (e.g., [45]–[47]), projections from the PFC may regulate anticipatory reward activity in the VS. In fact, one model of adolescent brain development suggests that later maturation of the PFC should increase activation to rewards in the striatum [5]. The data presented here and in other experiments are not consistent with this view, since adults did not activate the PFC more than adolescents. Notably, however, mesial PFC activity did not increase for either group during anticipation in the current modified MID task, nor does its activity increase during anticipation in the original MID task [29]. Consequently, the lack of age differences in mesial PFC activity during reward anticipation may reflect parameters unique to MID task design. Alternate tasks may thus have a different impact on age differences in prefrontal activity.

Although the hypotheses focused on the VS, exploratory whole brain analyses revealed that adults engaged a number of regions during anticipation of absolute reward that were not evident in adolescents. Direct comparison of groups revealed that adults showed greater activity during anticipation of absolute rewards in the cuneus, a component of extrastriate visual cortex. Extensive work suggests that motivationally relevant stimuli versus neutral stimuli modulate visual cortex activity [48]. For instance, reward magnitude increases activity in the cuneus and other regions [49]. However, modulation of cuneus activity may not only reflect value processing, since a recent study reported that both reward value and saliency modulated activity in the cuneus [50]. Further, adults, compared to adolescents, showed greater activity in the precuneus when processing large vs. small rewards [51]. Since the precuneus has been implicated in visuospatial processing [52], these findings suggest that adults may engage in deeper visuospatial attention to, or processing of, relatively larger reward cues, possibly reflecting greater adult sensitivity to the tactical or instrumental demands of the MID task. (We thank an anonymous reviewer on an earlier version of this manuscript for this interesting suggestion.) Notably, some regions were active in adults but not adolescents in the single group analyses that ultimately did not significantly differ in the direct group comparison. The current sample size (n = 18 per group) may have conferred insufficient power to detect significant group differences.

### Associations of individual differences in impulsivity with anticipatory activity

Theorists have long associated dopamine modulated reward circuitry with individual differences in Impulsivity and associated externalizing disorders [53], [54]. Although neuroimaging studies have demonstrated associations between activity in frontostriatal circuits and Impulsivity [23]–[26], [55], few studies have focused on links between reward anticipation in the VS and specific psychometrically-defined components of Impulsivity. Based on earlier psychometric analyses [3], [32], we selected two key aspects of Impulsivity: A_Imp and S_Imp. Both components were correlated with reward magnitude-related VS activity, such that higher Impulsivity scores (after controlling for age group) were associated with less VS differentiation between anticipation of medium versus small rewards.

The negative relationship between Impulsivity and VS activity is somewhat surprising given the predictions of earlier theories and research that links Impulsivity with increased approach motivation and sensitivity to rewarding stimuli [18], [25], [53], [54]. One possible explanation is that highly impulsive individuals are sensitive to rewards in general but insensitive to the differential value of medium versus small rewards. On the other hand, the Impulsivity-reward association is potentially complex and a number of recent fMRI studies have found negative associations between Impulsivity and VS activity during reward anticipation [23], [24], [56]. Analysis of dopamine gene variants and receptor binding studies using PET ligand imaging indicate that individual differences in Impulsivity are also associated with D2/D3 receptor availability in the striatum [55], [57]. Additionally, lower D2/D3 receptor availability has been associated with individual differences in Achievement [57], a measure with conceptual similarity to the A_Imp dimension in the present study.

Some of the inconsistencies across studies may be related to the specific Impulsivity scales used. Impulsivity is a heterogeneous construct that includes several underlying dimensions or components [32]. Furthermore, some measures that relate to Impulsivity (e.g., “sensation seeking”) also correlate with other dimensions of personality such as Extraversion or Positive Emotionality [32]. These “hot”, emotionally charged Impulsivity measures clearly tap distinct aspects of Impulsivity than the Impulsivity dimensions examined here. However, isolating distinct dimensions of Impulsivity is difficult given their overlap with other dimensions of personality [3], [32], [53]. Taken together with age group differences, these findings are consistent with the notion that developmental shifts in Impulsivity are linked to maturation of VS-mediated responses during reward anticipation. Future studies will, however, need to investigate associations between VS activity and Impulsivity using longitudinal designs capable of better characterizing growth trajectories. For instance, a recent two-wave longitudinal study found that increases in VS activity to peer facial expressions of emotion from Time 1 to Time 2 was linked to reduced susceptibility to negative peer influences and risky behavior [58].

Neither component of Impulsivity correlated with activity during anticipation of relative reward in the VS. Thus, whereas age differences are evident in absolute reward conditions and net differences in VS activity during anticipation of medium versus small rewards correlate negatively with Impulsivity, the relative reward effects (which showed some differences between age groups) show less of a relationship with Impulsivity. Interestingly, the correlation between Impulsivity and activity to large versus medium reward was not significant although trending in the opposite direction (see Table 1). Future studies with larger sample sizes will need to determine how activity to differing reward levels discriminates between high and low impulsive subjects.

### Strengths, limitations, and conclusions

This study featured several strengths, including a modification of a well-established design to study absolute as well as relative neural responses to anticipated reward and examination of the association of individual differences in Impulsivity with these neural responses, both within and across age groups. But this study also has some limitations. First, money was used as a reward. Although adolescents clearly value and report affective responses to monetary incentives, developmental differences in brain activity to monetary reward cues may be influenced by their relatively limited experience with purchasing goods and saving money. In the present study, although adolescents showed diminished anticipatory reward activation in the VS compared to adults, outcome related activity in the mesial PFC was generally similar to adults. Thus, it seems unlikely that lack of experience with money can completely account for differences in brain activity. Nevertheless, future developmental reward valuation studies may benefit from making the value of each reward more explicit by allowing subjects to use the money won from the task to purchase items that range in subjective value for each age group. Second, modifying the MID task to examine differences in relative reward processing made it possible to place the present findings in the broader context of other studies utilizing the original MID task [9], [14]. A simultaneous strength and limitation of the MID task is that subjects do not choose between different options. Thus, although reward anticipation is a critical component of decision-making [59], the present study cannot clarify whether the different activation profile shown by adolescents would generalize to other tasks that involve choice. Future studies will need to examine how relative reward processing influences choice in adolescents and adults. Third, since some forms of working memory continue to develop during the early teen-age years [60], reduced working memory capacity may have contributed to reduced relative reward activation differences in adolescents. Adolescents may have reduced awareness of the specific rewards that are in play on a given trial block and therefore show to similar activation during the anticipation of $1 reward regardless of context. On the other hand, both absolute and relative MID tasks are designed to minimize explicit working memory demands and rarely elicit activation during anticipation in brain structures commonly associated with working memory (e.g., the dorsolateral PFC). Future studies assessing relative reward valuation in adolescents may benefit from explicitly probing cue meaning during different trial blocks. Importantly, although absolute anticipatory reward processing does not require attentional and mnemonic tracking of block-to-block changes, adolescents still showed reduced anticipatory activation to absolute rewards. Thus, reduced working memory capacity cannot completely account for the overall picture of reduced anticipatory reward sensitivity in adolescents. Fifth, while targeted ROI analyses generally supported group differences in VS activity during reward anticipation, these differences were not as evident in whole brain analyses using cluster size corrections, suggesting that future studies seeking to show group differences may benefit from larger group sizes.

Despite these limitations, the results make a number of important contributions concerning the development of reward processing in the adolescent brain. Whereas previous studies have focused on whether adolescents show increased or decreased neural responses to rewards, the present study focused on more subtle differences in adolescent reward processing by targeting anticipation of both absolute and relative rewards. This sort of investigation may help reconcile the different findings of several studies and provide a developmental framework for identifying maturational processes that contribute to effective reward based behavior and choice in adulthood.
